# Matter-Aggregating Low-Dimensional Nanostructures at the Edge of the Classical vs. Quantum Realm

**DOI:** 10.3390/e25010001

**Published:** 2022-12-20

**Authors:** Adam Gadomski, Natalia Kruszewska

**Affiliations:** Institute of Mathematics and Physics (Group of Modeling of Physicochemical Processes), Faculty of Chemical Technology and Engineering, Bydgoszcz University of Science and Technology, Kaliskiego 7 Street, 85-796 Bydgoszcz, Poland

**Keywords:** nonequilibrium mesoscopic thermodynamics, nanostructure formation, reduction of system’s dimensionality, entropy production, quantum vs. classical, weak Van der Waals interaction, recuperation, stochastic quantization, Fokker–Planck type equation

## Abstract

This commentary tackles the subtle at-the-edge problem of passing locally by a mesoscopic matter-aggregating system from a classical stochastic to a quantum stochastic description. A *d*-dimensional entropy-productive aggregation of the matter is taken as the starting point. Then, a dimensional reduction towards a one-dimensional quantum-wire type matter-aggregation system is proposed, resulting in postponing surface-tension conditions for the effectively *d* = 1-dimensional quantum-wire type or nanorod-like cluster/polycrystal, which is qualitatively consistent with a physical-metallurgical (high-temperature) Louat’s grain growth model. A certain recuperative interplay based on maneuvering between subtle temperature rises applied to the system under study while maintaining its quantum character (the so-called Nelson’s quantum-stochastic procedure) within the limits of a vanishing Planck’s constant, involved in the diffusivity measure of the aggregation, is discussed. Certain applications towards the formation of *d* = 1-dimensional semiconductors and other nanostructures (possibly using soft materials or (bio)polymeric materials such as nanofibers) are envisioned. As a special example, one may propose a nanotechnological process which is termed the Van der Waals heteroepitaxy. The process itself contains the main quantum vs. classical crossover due to the involvement of weak repulsion (quantum) vs. attraction (treated classically) interactions, which are represented by a Lennard-Jones-type potential.

## 1. Introduction

It is commonly accepted that physical systems can uniquely express their matter aggregation properties if they are formed distinctly either within low-temperature (quantum prone; towards crystal formation) or within high-temperature (classically meant; towards amorphization) limits. What appears at the edge, just between quantum and classical manifestations, is often shifted to an exploration of the respective phase transitions the systems may undergo. For example, if the first-order phase transition scenario occurring between the corresponding thermodynamic equilibria comes into play, then a clear distinguishment and the resulting coexistence of the emerging phases applies, for example, as in the case of a triphasic water system or biphasic (ferro)magnetic/electric system (second-order phase transition), to mention but two.

**Figure 1:**
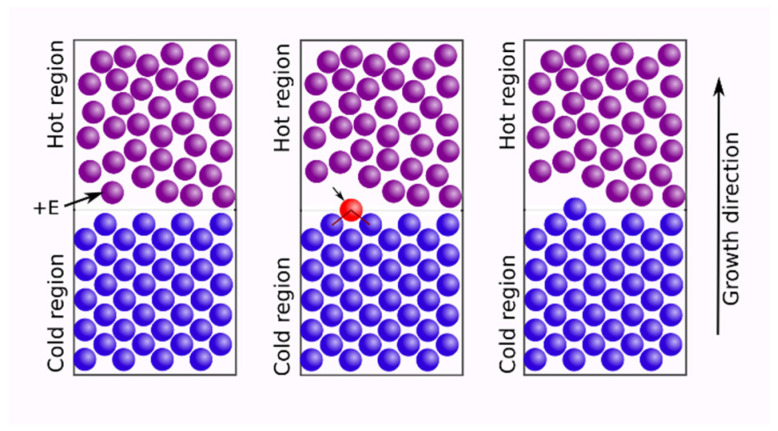
Schematic two-dimensional cross-section of a two-state slab-like nanosystem differing locally with temperature. There is a possibility that one of the atoms or molecules from the hot region (marked by red) will gain a “quantum” of thermal energy, *E*, to travel over the diffuse boundary into a colder region of greater structural order. It thereby loses this additional energy by, for example, bond creation, a quantum-mechanical effect, thereby causing orderly growth of the colder grain.

In what follows we wish to exploit a certain non-phase-transitive way of creating a matter-aggregation output, the parts of which, while slightly out of equilibrium, benefit from a type of recuperation of its apparently contradictory properties. This refers to being at the edge of the fundamental quantum-classical thermal passage(s), meaning that a subtle (non-drastic) temperature drop/rise can eventually cooperate with the onset of the corresponding quantum behavior [1]. The overall scenario undergoes a type of recuperation process in which a subtle local rise in temperature in a neighboring part of a material piece causes its constituents, such as atoms or molecules, to pass over a diffuse interface or a boundary to its low-temperature vicinal counterpart (see, Figure 1) without experiencing a pressure difference between these parts but rather because of being trapped by the low-temperature part due to a local bond creation or similar. Such a self-organizational effect is especially foreseeable if there is a certain loss in surface-tension influence caused by a reduction in dimensionality of the said material’s piece, presumably to an effective space dimension approaching one, as in the case of quantum wires and/or nanorods, or (bio)polymeric nanofibers [2] and similar materials.

Materials science and condensed-matter literature, especially that pertinent to nano- and semiconductor technology, but also that concerning modern polymer- metal- or composite technologies, contains a good number of such examples (See [1,2,3]).

In what follows, we would like to briefly discuss a mesoscopic, entropy-productive material formation based on an evolution of grain containing systems, wherein the grain’s volume is a stochastic variable (*x*). The material system addressed is supposed to sit close to the local thermodynamic equilibrium, eventually escaping from it for another one. The escape is assumed because a local thermal agitation applies to the system, let us say, by a local temperature gradient.

Next, when realizing how such material evolutions proceed, we wish to confine ourselves to a practically relevant circumstance (towards quantum wires or low-dimensional nano-object formations) of effectively reaching the space dimension *d* = 1. As a consequence, our system is assumed to be systematically devoid of the role customarily played by surface tension. However, to keep the needle-like system as a whole, we foresee that the Van der Waals attraction ought to be at play while creating the structure. On the other hand, it qualitatively appears we would confine it to a line (or a chain) of a bubble-containing system or apply a similar procedure [4].

At the same time, we assume that the system, still possessing some remnant surface tension contribution due to being not ideally one-dimensional in terms of geometry, would lose this surface tension if moved onto a higher temperature (amorphization or disorder) level [5]. In this way, a one-dimensional diffusion equation for the evolution in the *x*-space is obtained [6].

Further on, it is allowed that the respective high-temperature limit permits the quantum effects to work locally, as in chemical bond creation-annihilation events. A stochastic quantization procedure applied to diffusion-type systems, first proposed by Nelson [7], and elaborated later by Ruggiero and Zanetti [8] in terms of a quantum-classical crossover in critical dynamics, allows for converting the diffusion equation (in general, equations of the Fokker–Planck or Smoluchowski types) to a Schrödinger’s equation (in the imaginary time domain). This is plausible if the diffusion coefficient is proportional to Planck’s constant *h* [7,8]. With the limit of *h* tending to zero, i.e., while escaping from the respective quantum realm, the material evolution in *x*-space will approach a low-valued ‘subdiffusive’ (and, non-quantum) pathway, nearly causing the atoms or molecules entering any of the low-temperature adjacent grains or clusters to “arrest”. Of course, such a rationale is proposed when based on a mesoscopic nonequilibrium-thermodynamic, grain-containing system [5,9], but its realization can be performed with the incorporation of local quantum-classical effects, which in general, leads to the recuperation of the atoms’ (or, molecules’) thermal energies. These are increased in one grain/cluster and then used to create a polycrystalline order when the thermally excited atom or molecule becomes a part of an adjacent ‘cool grain’ (cf. Figure 1). We are of the opinion that such atoms’ (or, molecules’) co-optation mechanism is not frequently taken into account in modern contemporary nanotechnologies [1,2,3].

The article is structured as follows. In Section 2, a mesoscopic model for a material formation in nonequilibrium-thermodynamic conditions is described, and in Section 3, the classical-quantum limit/crossover of the *d* = 1-dimensional clusters formation is revealed. Section 4 consists of a related discussion and a summary.

## 2. Mesoscopic Model for Material Formation in Nonequilibrium Thermodynamic Conditions

Here below, let us sketch in brief a model of the normal grain growth that is based on a decomposition of the matter flux *J*(*x*,*t*) into two main parts [9].

(1) $$J\left(x,t\right)=-\sigma x^{\alpha -1}f\left(x,t\right)-Dx^{\alpha }\frac{\partial f\left(x,t\right)}{\partial x}.$$

Namely, a surface tension involving the non-gradient part and its counterpart with a Fickian-type gradient. Note that *σ* and *D* are surface tension and diffusion reference parameters, respectively. The function *f*(*x*,*t*) stands for the probability density of finding the respective grain of size *x* at time *t*. The exponent α reads:

(2) $$\alpha =1-\frac{1}{d}\;,$$

where *d* is the Euclidean space dimension (*d* = 1, 2, 3…). Bear in mind that if *d* = 1, then *α* = 0, and in Equation (1), the Fickian-type term becomes classically defined with a constant *D*. By virtue of this, the factor preceding the gradient of *f*(*x*,*t*) becomes *x*-independent.

Let us assume that, as in [4,5,9], the system is conservative, which means that

(3) $$\frac{\partial f\left(x,t\right)}{\partial t}+\frac{\partial J\left(x,t\right)}{\partial x}=0\;.$$

To complete the problem presented in Equations (1)–(3), one has to prescribe the initial and boundary conditions (IBCs). The so-called delta-Dirac and absorbing IBCs can be found elsewhere [9].

This phenomenological model, most likely first invented by Louat [6], for some physical-metallurgical grain-containing system evolving diffusively in the so-called normal grain-growth conditions (see the mention of IBCs above) is an entropy-production model. According to [5,9], it can be presented (compare with Equation (1)) in dimension *d*. In terms of the entropy production, the matter flux is given by [9]

(4) $$J\left(x,t\right)=-b\left(x\right)f\left(x,t\right)\frac{\partial \varphi \left(x\right)}{\partial x}-Dx^{\alpha }\frac{\partial f\left(x,t\right)}{\partial x},$$

**Figure 2:**
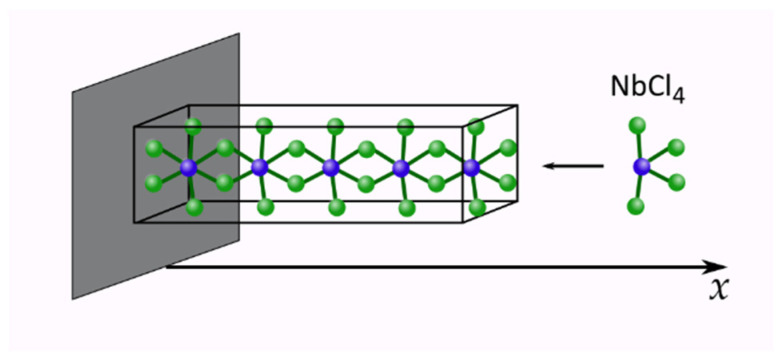
One-dimensional growth of quantum wire on the NbCl_4_ example. This material has strong covalent bonds in the *x* direction (in accordance with the direction of the atomic chain). The remaining bonds are weaker, being of the Van-der-Waals type [12] (The horizontal *x* direction is taken arbitrarily here).

In this case, the only difference, when comparing Equations (1) and (4) is that there appears a free energy gradient $\frac{\partial \varphi \left(x\right)}{\partial x}$, which implies that the coefficient (mobility) $b\left(x\right)=\frac{1}{Tf\left(x,t\right)}L\left(x\right)$, with *L*(*x*), an Onsager’s coefficient [9]. The most important physical fact here appears to be that *b*(*x*) is inversely proportional to the local temperature *T* (slightly changing around its mean value, pointing to the content of Figure 1); thus, for the high-temperature limit, the non-Fickian term, represented by the free energy gradient ($\frac{\partial \varphi \left(x\right)}{\partial x}$), vanishes irrespective of the role played by dimension *d*. Following the fact that the Van der Waals forces between atoms and molecules can be modeled as fluctuating electric dipole oscillators, as described by the classical harmonic oscillator equations [8], which involve masses coupled by springs, we can take the free energy gradient in the form of the spring force ($-\frac{\partial \varphi \left(x\right)}{\partial x}$, which is of the form −*kx*, with *k* being a constant). Based on this, we can deduce that the presented model conforms to Van der Waals when k→0, which is true for most of the materials at high temperatures (T→∞). If *φ* represents the elastic energy of a 1D microslab, the material spring constant involved in this kind of energy ought to attain a negligible value, which is the situation of a system equipped with a set of weak bonds, as is expected to occur in Van der Waals oxide heteroepitaxy [10,11]. As expected, the evolution of the grainy (“granular”) matter in one-dimensional *x*-space becomes fully Fickian, as was shown in [5]. However, bear in mind that we claim that in the exemplary heteroepitaxy just invoked, an effective one-dimensional diffusion component is the feeding mechanism for the formation of needle-like domain-wise structures; see [11] and Figure 3 therein (cf., Figure 1). It is based on the flux:

(5) $$J\left(x,t\right)=-D\frac{\partial f\left(x,t\right)}{\partial x},$$

where *D* = const, which is independent of the state variable *x*. Of course, the evolution/formation of the *d* = 1-material object remains conservative, according to Equation (3) for α = 0, which is equivalent to *d =* 1, (cf. Equation (2)). This is very likely when the formations of quantum wires during a molecular beam (hetero)epitaxy process, or similarly, when by the same technology an emergence of nanorods becomes effective [1,2] or some nanofibrils and their bundles appear as one-dimensional Van-der-Waals quantum materials [3,12] (cf. Figure 2). The scientific interest is growing in the latter as the quantum material’s specific *d* = 1-structural characteristics lead to highly anisotropic optical and electrical properties, which is advantageous for many applications [12].

To be specific, let us refer partly to the example(s) presented in [12], and visualized in Figure 1 and Figure 2. Thus, we may offer our physical scenario that reveals the recuperative phenomenon of interest (cf. Figure 1). The phenomenon, actually a nanotechnological process, is termed the Van der Waals (oxide) heteroepitaxy, as already mentioned above. It relies on a controlled diffusion formation [10,11,13,14], which we simplify to the effective one-dimensional process. For some obvious reasons, pointing to geometrical (V type grooves) [13] and catalytical factors (CCl_4_ environment [14]) readily influencing the process, making it one-dimensional, our model is capable of capturing the needle-like quantum-wire involving formation. Based on Figure 1, one can assume that after the diffusion-controlled deposition of atoms/molecules, two types of structural domains appear. The domain emerging closer to the source of deposition can be recognized as a hot or disordered domain, whereas the more distant domain is more ordered and can be viewed as the cold one (cf. Figure 1). While the main atomic/molecular construction of the quantum wire retains its elasticity, and non-affinity to the deposition (vicinal) surfaces, other atoms or molecules of the construct are held to the “elastic skeleton”, and to themselves, by weak Van der Waals forces. For them, the descriptive Lennard-Jones (L-J) potential includes its repulsive character (quantum type, with Pauli principle involved); thus, it is proportional to $1/r^{12}$, with *r* being a binary interaction distance, but it also contains the attractive term (viewed classically as $\sim 1/r^{6}$), which is temperature dependent and assures the attraction at longer distances. This L-J potential per se includes a quantum-classical paradigm, very suitable for our viewpoint. In what follows, we offer our line of argumentation in this respect while maneuvering the near distance *r* close to the L-J energy minimum.

## 3. Classical vs. Quantum Limit of the *d* = 1-Dimensional Cluster Formation

As we have already arrived at a one-dimensional Van-der-Waals type formation of materials in terms of appropriate space dimension reduction for and suitable high-temperature application to the clustering system [5,6], we can also strive to achieve the formation quantum. This can be formally achieved thanks to a quantization procedure first proposed by Nelson in the 1960s [7]. In this procedure, a derivation of Schrödinger’s equation can essentially be obtained from the Newton–Langevin type dynamics, with the incorporation of Ornstein–Uhlenbeck noise, mimicking an impact of the external environment on the system’s dynamics. It appears, for example in [13,14], that the selection of a nonreactive substrate based on a mica-type catalyst and the incorporation of a suitable growth-amplifying environment play exclusion (towards 1D) roles. From Nelson’s quantization procedure, also thoroughly elaborated in [8], it turns out that the diffusion coefficient included in Equation (5) obeys

(6) D∼h

if we make use of, as already introduced before, an effective analogy between the random walk in the *x*-space of grain sizes (effective grain lengths; “needles”) and conventional (for Brownian particles) coordinates on the straight line (*d* = 1) [15], (cf. the kinematics of Markov process described in [7]) by means of Brownian motion of a particle which also involves an inverse proportionality to the particle’s mass (*m*) [8], i.e., D~1/m. Of course, the Planck’s constant, *h*, viewed in dimensions of action (formally, Joule times second) is known as a very small quantity. It is also well-known that turning the evolving system to the classical limit implies that

(7) h→0

would mostly apply, which is surprisingly consistent with the similarity expressed by Equation (6). It also refers to the fact that the diffusion of grain boundaries associated with the recuperation events, though normal, becomes very small along the interdomain (dividing, but fuzzy) surface (cf. Figure 1). Moreover, the negligible role played by the surface tension in the 1D case is replaced in our argumentation by an effective involvement of the attractive part of the weak Van der Waals interactions. The repulsive counterpart of this interaction assures the atom-exclusion effect, which is well supported by the Pauli electron-orbitals exclusion principle. Both Equations (6) and (7) applied to the quantum-classical crossover of the systems (Fokker–Planck type) dynamics [7,8,15] are going to witness the physical fact the atoms or molecules (or similar) when migrating from the hot grain to its subtly cooler adjacent counterpart (cf. Figure 1), will be capable of performing there a very slow motion. This can be described as a quantum-diffusive (limited by bonding) random walk [16] within the cooled grain/cluster or when trapped in a challenging time domain within a soft granule [17]. A general discussion on classical-quantum crossover can be found in [18].

## 4. Discussion and Summary

In this paper, we suggest that a mesoscopic nonequilibrium-thermodynamic system, by its dimensionally-reduced evolution, is capable of absorbing a quantization procedure that makes the uncovered quantum-classical crossover dynamics effective and fairly unambiguous. A certain relevant physical fact, pertinent to the realization of the (quantization [7,8]) procedure, is that some additional (auxiliary) sub-procedure has to be performed on the diffusion coefficient’s value and its connection with the Planck’s constant (*h*) (cf. Equations (6) and (7)), and thus with the quantum world. This was proposed in a similar vein and discussed some years ago for another low-dimensional biopolymer system [19]. The exemplary nanotechnological process is termed the Van der Waals (oxide) heteroepitaxy, resting essentially, in terms of our rationale, on a controlled fed-by-diffusion formation which has been reduced to an effectively one-dimensional picture, in which surface-tension effects have been virtually replaced by Van-der Waals interactions.

The elementary entropy change $\delta S=-\frac{1}{T}\int \mu \left(x,t\right)\delta f\left(x,t\right)dx$, as provided by [9], depends upon the chemical potential of the system *µ*(*x*,*t*), which in turn, has to depend upon the (elementary) free system’s energy and a chemical affinity [20]. We believe that we have to attribute δS as a useful cause of the recuperation of the warmer units (atoms, molecules, hydrogen-bonding oligomers [21,22]) being absorbed by their adjacent cooler counterparts. In this manner, an effective *d* = 1-structure can form, and repetitive realization of *µ*(*x*,*t*) would rely on the above described recuperative units’ elementary exchange processes. The chemical affinity, in turn, is likely realized by means of bonding, for example H-bonding, as described in [23]. It can also challenge self-organizationally an affinity of the effectively one-dimensional atomic (*Ge*) object to the substrate (*Si*) on which it is grown [24].

## Data Availability

Not applicable.
